# Enhanced aphid abundance in spring desynchronizes predator–prey and plant–microorganism interactions

**DOI:** 10.1007/s00442-016-3768-1

**Published:** 2016-11-17

**Authors:** Benjamin Fuchs, Tatjana Breuer, Simone Findling, Markus Krischke, Martin J. Mueller, Andrea Holzschuh, Jochen Krauss

**Affiliations:** 1 Department of Animal Ecology and Tropical Biology, Biocenter, University of Würzburg, Am Hubland, 97074 Würzburg, Germany; 2 Department of Pharmaceutical Biology, Biocenter, University of Würzburg, Julius von Sachs Platz 2, 97082 Würzburg, Germany

**Keywords:** Multi-trophic interactions, Pest control, Herbivory, Insect timing

## Abstract

Climate change leads to phenology shifts of many species. However, not all species shift in parallel, which can desynchronize interspecific interactions. Within trophic cascades, herbivores can be top–down controlled by predators or bottom–up controlled by host plant quality and host symbionts, such as plant-associated micro-organisms. Synchronization of trophic levels is required to prevent insect herbivore (pest) outbreaks. In a common garden experiment, we simulated an earlier arrival time (~2 weeks) of the aphid *Rhopalosiphum padi* on its host grass *Lolium perenne* by enhancing the aphid abundance during the colonization period. *L. perenne* was either uninfected or infected with the endophytic fungus *Epichloë festucae* var. *lolii*. The plant symbiotic fungus produces insect deterring alkaloids within the host grass. Throughout the season, we tested the effects of enhanced aphid abundance in spring on aphid predators (top–down) and grass–endophyte (bottom–up) responses. Higher aphid population sizes earlier in the season lead to overall higher aphid abundances, as predator occurrence was independent of aphid abundances on the pots. Nonetheless, after predator occurrence, aphids were controlled within 2 weeks on all pots. Possible bottom–up control of aphids by increased endophyte concentrations occurred time delayed after high herbivore abundances. Endophyte-derived alkaloid concentrations were not significantly affected by enhanced aphid abundance but increased throughout the season. We conclude that phenology shifts in an herbivorous species can desynchronize predator–prey and plant–microorganism interactions and might enhance the probability of pest outbreaks with climate change.

**Electronic supplementary material:**

The online version of this article (doi:10.1007/s00442-016-3768-1) contains supplementary material, which is available to authorized users.

## Introduction

Climate change causes shifts in the timing of seasonal events (phenological shift) in many species (Van der Putten et al. 2010; Stevenson et al. 2015; Thackeray et al. 2016). In a climate change meta-analyses on 677 species, 62% of species shifted their phenology towards spring advancement, while 38% of the species did not shift or shifted even towards a delay of spring events (Parmesan and Yohe 2003). Such different responses to climate change can cause desynchronizations between interacting species which can lead to a breakdown of mutualistic interactions (Kiers et al. 2010). Climate change driven shifts in species phenology are expected to impact species abundances and interactions (Tylianakis et al. 2008), as shown, e.g., in a disruption of predator–prey dynamics due to climate driven temporal desynchronization of peak abundances between prey and predators (Visser et al. 2006). The majority of studies on phenological mismatches have focused on just two trophic levels, while studies considering three or more species levels are rare (Rafferty et al. 2013). Primary consumers are more susceptible to climate change than primary producers and secondary consumers which can cause mismatches along trophic cascades (Thackeray et al. 2016). In two laboratory experiments on multi-trophic interactions involving insect crop pest species, higher temperature caused an increase in aphid abundance (Bezemer et al. 1998; Marquis et al. 2014). In parallel, host plant biomass decreased and top–down control by parasitoids increased in one study (Bezemer et al. 1998), whereas predator abundance decreased in the other study (Marquis et al. 2014). Despite the importance of such studies for biological pest control under climate change, similar studies under more realistic field conditions are missing.

Aphids are good model organisms to study multi-trophic interactions (Härri et al. 2008). Many aphid species are serious crop pests that cause damage by feeding on plant sap or act as vectors of virus diseases (Van Emden and Harrington 2007). Phenotypic plasticity, rapid growth rate, and multivoltine life cycles are advantageous for aphids responding to climatic changes (Ward and Masters 2007). Due to their parthenogenetic reproduction during the summer months, aphid populations can grow exponentially (Costamagna et al. 2007). First flight trends of aphids advanced in the last five decades (Bell et al. 2015) and will further advance by ~8 days per 1 °C higher early spring temperature (Harrington and Clark 2010). Aphid populations are top–down controlled by predators or parasitoids (Schmidt et al. 2003; Chen 2008), but in contrast to aphids, predators have lower population growth rates and 5–20 times longer development times than aphids (Snyder and Ives 2003), which might be a constraint in responding to climate change (Reed et al. 2011). Furthermore, primary consumers, such as aphids, showed higher climate sensitivity compared to secondary consumers (Thackeray et al. 2016).

Aphid populations are not only top–down controlled, but can also be bottom–up affected by chemical defense mechanisms of plants or plant-associated microbial symbionts (Müller and Krauss 2005; Chen 2008). Seedborne fungal endophytes infecting aerial parts of cool-season grass species can play an important role in plant defense against herbivores (Clay 2014), but see (Saikkonen et al. 1998; Cheplick and Faeth 2009). Vertically transmitted fungal grass–endophytes of the genus *Epichloë* affect the grass physiology depending on biotic and abiotic conditions by increasing biomass (Müller and Krauss 2005), enhancing drought resistance (Hesse et al. 2003), or altering plant compositions (Rudgers and Clay 2007). *Epichloë* endophytes produce herbivore toxic alkaloids in the plant, which decrease the fitness of invertebrate herbivores or cause intoxications of grazing livestock (Müller and Krauss 2005; Schardl et al. 2013). On the other hand, it is under debate whether endophyte and alkaloid concentrations increase with insect herbivory (Hartley and Gange 2009; Zhang et al. 2009; but see Fuchs et al. 2016). Grazers can drive plant–endophyte dynamics which can lead to higher infection levels in grass populations with high herbivore pressure (Koh and Hik 2007).

Our field experiment contained four interacting species along a tri-trophic level food chain (1) grass endophyte (*Epichloë festucae* var. *lolii*), (2) host plant (*Lolium perenne*), (3) aphid (*Rhopalosiphum padi*), and (4) aphid predators. We investigated the effects of enhanced aphid abundance in spring on aphid population development, predator occurrence and abundance, plant biomass, and endophyte growth and alkaloid production. The key question of this study is whether organisms that interact with aphids have the phenological plasticity to respond to the simulated aphid shift. Fertilizer is often used on grasslands with our host plant *L. perenne,* and fertilization can enhance plant growth, endophyte-derived alkaloids, and abundances of aphids and their predators (Gastal and Nelson 1994; Krauss et al. 2007). We, therefore, included a fertilizer treatment in our study to verify our results for differently fertilized grasslands.

Our main predictions are:

1. Experimental setting: enhanced aphid abundance in spring (aphid shift) leads to overall higher aphid abundances.
2. Top–down control: enhanced aphid abundance in spring (aphid shift) leads to a desynchronization between aphid and predator phenology.
3. Induced defense: endophyte and alkaloid concentrations increase in the host grass due to enhanced aphid abundance in spring (aphid shift).
4. Bottom–up control (endophytes): aphid population growth is reduced on endophyte infected plants, due to the production of insect toxic compounds.

## Materials and methods

### Experimental design

In a common garden experiment with a randomized block design, we tested the effects of (1) enhanced herbivore abundances in spring (aphid: *Rhopalosiphum padi*), (2) endophytic fungus infection (*Epichloë festucae* var. *lolii*) in host plants (*Lolium perenne*), and (3) fertilization of host plants on trophic interactions along a food chain. Every treatment combination was represented once per block resulting in eight randomly arranged pots per block, and replicated ten times. A block design was chosen to control for unexplained variances if block has a significant influence. Altogether we used 80 pots (18 × 18 × 18 cm) with common garden soil (Einheitserde classic CL ED73, Profi Substrat) with a nitrogen availability of 250 N mg L^−1^. Each pot was sown with 10 *L. perenne* seeds of the grass cultivar Samson at the end of March 2013. Seeds were provided by David Hume, AG Research, NZ. The same cultivar was used in previous experiments and alkaloid studies, including aphids and aphid predators (Meister et al. 2006; de Sassi et al. 2006; Fuchs et al. 2013, 2016). In 40 pots, the seeds were infected with the endophytic fungus *Epichloë festucae* var. *lolii*, which is formerly known as *Neotyphodium lolii* Glenn, Bacon, and Hanlin (identity number A 12038) (Leuchtmann et al. 2014). In 40 other pots, seeds were not infected by the endophytic fungus (identity number A 11104). In the following, we abbreviate *E. festucae* var. *lolii* infected plants with “E+” and uninfected with “E−”.

After 40 days of rearing the plants in a greenhouse, plants were transferred to the field, where the distance between grass pots was 30 cm to avoid contact between plants. In the field, in half of the pots, aphid abundance was experimentally increased (simulated phenology shift see below), and half of the pots were treated with additional fertilizer in a crossed design. We used NPK fertilizer (Compo 20-5-10) equivalent to 400 N kg ha^−1^ year^−1^ in eight doses between May 15th and July 3rd. We abbreviate fertilized plants with “F+” and non-fertilized with “F−”.

The study site was fenced to exclude vertebrate herbivores, mainly rabbits. We trimmed the plants three times at the beginning of the field experiment to a height of 20 cm to avoid contact to surrounding plants. We finished the field experiment at 14th of August 2013 with taking the aboveground biomass of the plants at an age of 140 days. Only aboveground biomass was taken, as *E. festucae* var. *lolii* only infects aerial plant parts (Cheplick and Faeth 2009).

### Aphid addition

Grass plants were 56 days when we started our experiment on May 22nd by adding 60 adult aphids *Rhopalosiphum padi* in each of 40 pots (Aphid supplier: Katz Biotech http://www.katzbiotech.de). Due to a low survival rate of the added aphids due to the changeover from rearing conditions in the supplier’s lab to common garden conditions, another 60 aphids per pot were added on June 4th. *R. padi* aphids overwinter on the bird-cherry tree and change their host in spring to cereal plants (Dixon 1971). We were checking grass plants daily for natural aphid arrival from beginning of May to have exact dates to adjust our aphid addition. As aphid arrival is not exactly predictable, an actual shift of 2 weeks earlier was not possible but our enhanced aphid abundances in spring simulated an advancement of aphid phenology compared to natural aphid phenology by ~2 weeks (treatment abbreviated as “A+”). We achieved this by (1) recording the time of natural occurrence of single individuals of *R. padi* on our pots; (2) we estimated that 2 (to 3) adult aphids occur 2 weeks earlier when they shift their phenology; (3) we estimated the overall fecundity of *R. padi* with ~30 offspring within 2 weeks averaged for endophyte free and endophyte infected host grass of the cultivar Samson (Meister et al. 2006); and finally, (4) we estimated a 50% mortality of added aphids caused by the changeover from rearing conditions in the laboratory to outdoor conditions (personal observations). We chose to simulate 2 weeks, as it represents an aphid shift with an increasing spring temperature by ~2 °C which represents a range between the SRES climate change predictions for 2099 (IPCC WG III 2000; Harrington and Clark 2010). We are aware that climate warming can also change endophyte and host grass growth (Vega-Frutis et al. 2014; McCulley et al. 2014), but the focus of our study was to uncover effects of increased aphid abundances in spring on interacting trophic levels.

The other 40 grass pots received no additional aphids (treatment abbreviated as “A−”), but all 80 pots were exposed to naturally immigrating aphids under natural common garden conditions.

### Surveys

We counted aphids for 5 min per pot once a week for a total of 8 weeks during summer. We started at a plant age of 77 days at June 12th counting every 7 days up to a plant age of 126 days at 31st of July 2013 (calendar weeks 24–31). In few cases in the calendar weeks 26–28 5 min were not enough to count the whole pot. In such cases, only half or a quarter of the pot was counted and aphid numbers were extrapolated for the whole pot. Due to a symmetrical plant phenotype and a homogenous aphid distribution all over the plant, we multiplied the recorded aphid number by two (in case of counting half of the pot) or multiplication by four (in case of counting quarter of the pot). Predators of aphids, including hoverfly larvae and pupae, lacewing larvae, all ladybird stages, and spiders, were counted for 3 min per pot, which was always sufficient time to count the whole pot. After 8 weeks of the experiment, aphid abundance and predator abundance were too low for meaningful counts.

Plant samples to quantify endophyte concentration and alkaloid concentrations were taken in parallel to aphid and predator counts, but were taken for two additional weeks until a plant age of 140 days at 14th of August 2013 (calendar weeks 24–33). We collected plant material for quantitative PCR (qPCR) and Ultrahigh Performance Liquid Chromatography–Mass Spectrometry (UPLC-MS) analyses by cutting a 3 cm piece from the plant, around the oldest leaf sheath. Withered parts were removed from the sampled material to ensure similar sample quality. We sampled one tiller per pot per week of all 80 pots. After grounding the samples in liquid nitrogen, we split the grass material onto two tubes, one for UPLC-MS analysis and one for qPCR analysis. At the 14th of August, we harvested the total aboveground biomass of all pots. Biomass was dried for 3 days in a 60 °C tempered drying oven (Memmert GmbH) before weighing.

### Alkaloid extraction and analysis

We quantified the alkaloids peramine, lolitrem B, and ergovaline produced by the endophytic fungus, with peramine being the most insect deterring alkaloid (Tanaka et al. 2005). We sampled weekly about 3 cm plant material from leaf stalks and leave sheaths of endophyte infected and endophyte free *L. perenne* plants which was immediately frozen after sampling. We analyzed weekly taken plant material from E+ pots (sampling see above), while from E-pot samples were analyzed at the end of the study period to confirm that all E− pots are free from endophyte infection and alkaloids. We weighed the grass material with a microscale (Mettler-Toledo Intl. Inc.) before alkaloids were extracted from the samples with methanol and dichloromethane in several steps. Afterwards, alkaloids were determined and quantified with Ultrahigh Performance Liquid Chromatography (UPLC-MS) with argon as collision gas. Using our previous published UPLC-MS method developed to detect and quantify alkaloids produced by *E. festucae* var. *lolii* (Fuchs et al. 2013), we quantified the peramine concentration with reference to the internal standard compound homoperamine. Lolitrem B concentration was quantified semi-quantitative by reference to homoperamine. Ergovaline was quantified with the internal standard compound ergotamine. Detection limit for all alkaloids was 5 ng.

### Endophyte quantification by qPCR

Endophyte concentration was determined by quantitative PCR (qPCR) analysis (detailed protocol see (Fuchs et al. 2016; modified from Rasmussen et al. 2007). Genomic DNA (gDNA) was extracted from circa 50 mg powdered grass material. Exact sample weight was not needed, because we quantified the amount of amplified endophytic gDNA by reference to amplified grass gDNA per sample. For quantification of the endophyte gDNA, we used a fungal specific primer (Chitinase A), and for the plant gDNA quantification, we used a grass specific primer (β-tubulin).

All presented fungal gDNA results refer to 10,000 copies of amplified grass β-tubulin transcripts.

Uninfected samples showed up to 200 copies of fungus per 10,000 copies of grass β-tubulin transcripts which is caused by primer dimers and general background noise. Consequently, we set samples as endophyte free below 200 copies of fungal gDNA. Non-infection was confirmed by the absence of endophyte produced alkaloids, too.

### Statistics

All statistical analyses were conducted using the software R version 3.0.2. We used ANOVAs with the three explanatory variables aphid shift (A+/A−), endophyte infection (E+/E−) and fertilizer (F+/F−). The treatments were arranged in a randomized balanced block experiment (Crawley 2012), resulting in eight treatment combinations, which we replicated each ten times. Response variables were (1) plant biomass, (2) aphid abundance, and (3) aphid predator abundance. Total aphid and predator abundances were estimated by summing up the numbers of aphids and predators, respectively, per pot during 8 weeks of counting. Block was tested as a fixed factor in the models, but was never significant and never changed our results. We, therefore, simplified our models and omitted “block” from the analyses (Supplementary material) (Crawley 2012). None of the treatment interactions showed significant results (all *p* > 0.05) and were, therefore, omitted from the final models and are not further discussed. We also used ANOVAs to analyze separately the 40 pots with endophyte infected plants (E+) to detect the effects of aphid shift (A+/A−) and fertilizer (F+/F−) on (4) alkaloid concentrations and (5) endophyte concentration. We used mean of alkaloid concentration and endophyte concentration per pot during 10 weeks of sampling to test for differences between our treatments over the whole study period. We also analyzed ANOVAs separately for each week to detect if the endophyte concentration, aphid abundance, and predator abundance differ between treatments in single weeks. We did not apply repeated measure analyses, because it is not an appropriate method to detect differences in time but to correct for temporal pseudo-replication (Crawley 2012). Residuals in all models had homogenous variances and were normally distributed. Means ± standard errors are presented throughout the manuscript. Alkaloid concentrations over time were illustrated with a local polynomial regression fitting model (Cleveland et al. 1992).

## Results

We recorded 158,385 aphids on the 80 pots within the 8 week study period. *Rhopalosiphum padi* was with 98.57% the most dominant aphid species followed by *Aphis fabae* 1.38% and *Sitobion avenae* 0.05%. We considered only *R. padi* in our analyses, as we added this species to simulate phenology shift. A total of 1307 aphid predators were recorded during 8 weeks; however, none was detected in the first 2 weeks of our study. Hoverfly larvae (644 individuals) were the most abundant predators.

In total, we recorded 2.5 times more aphids per pot with aphid shift (A+) than on pots without aphid shift (A−) (Table 1; Fig. S1b). Endophyte infection and fertilization had no significant effect on aphid abundance (Table 1). Predator abundance was neither significantly affected by aphid shift nor by endophyte infection, but was higher on fertilized pots (Table 1; Fig. S1a). Aboveground plant biomass at the end of the experiment was higher in endophyte infected pots compared to uninfected pots and in fertilized pots compared to unfertilized pots (Table 1). Aphid shift increased the endophyte concentration in endophyte infected plants by 25% (Table 1, Fig. S1c), but did not significantly influence alkaloid concentrations (Table 1). Fertilization affected the lolitrem B concentration, but did not significantly affect concentrations of the alkaloids peramine and ergovaline (Table 1).

**Table 1:** ANOVA table showing the effects of aphid shift (enhanced abundance), endophyte infection, and fertilization on aphid abundance, predator abundance, and plant biomass; and of aphid shift and fertilization on endophyte concentration (gDNA × 10^4^ referred to 10^4^ copies of grass gDNA) and alkaloid concentrations (µg/g) tested only for endophyte infected host plants

Response	Predictor	*df*	*F*	*P*	Mean ± SE	Mean ± SE
All pots						
Aphid abundance	Aphid shift	1.76	38.60	**<0.001**	A−: 1152 ± 134	A+: 2888 ± 243
	Endophyte infection	1.76	0.64	0.43	E−:1908 ± 207	E+:2132 ± 269
	Fertilization	1.76	0.31	0.58	F−:1942 ± 263	F+:2098 ± 215
Predator abundance	Aphid shift	1.76	1.01	0.32	A−: 14.9 ± 1.38	A+: 17.2 ± 1.85
	Endophyte infection	1.76	0.83	0.36	E−: 15.0 ± 1.81	E+: 17.1 ± 1.44
	Fertilization	1.76	7.34	**0.007**	F−: 13.0 ± 1.88	F+: 19.1 ± 1.18
Biomass (g)	Aphid shift	1.76	0.99	0.32	A−: 41.6 ± 2.1	A+: 39.9 ± 2.0
	Endophyte infection	1.76	4.09	**0.046**	E−: 39.0 ± 2.1	E+: 42.5 ± 2.0
	Fertilization	1.76	146.11	**<0.001**	F−: 30.4 ± 1.3	F+: 51.1 ± 1.1
Only endophyte infected pots						
Endophyte conc.	Aphid shift	1.37	7.05	**0.012**	A−: 1.21 ± 0.06	A+: 1.62 ± 0.14
	Fertilization	1.37	0	0.99	F−: 1.41 ± 0.12	F+: 1.41 ± 0.11
Peramine (µg/g)	Aphid shift	1.37	2.75	0.11	A−: 3.94 ± 0.21	A+: 3.53 ± 0.13
	Fertilization	1.37	0	1	F−: 3.73 ± 0.17	F+: 3.73 ± 0.19
Lolitrem B (µg/g)	Aphid shift	1.37	0.06	0.80	A−: 1.87 ± 0.16	A+: 1.83 ± 0.09
	Fertilization	1.37	4.90	**0.035**	F−: 2.04 ± 0.14	F+: 1.66 ± 0.09
Ergovaline (µg/g)	Aphid shift	1.37	1.27	0.27	A−: 0.072 ± 0.005	A+: 0.082 ± 0.007
	Fertilization	1.37	0.85	0.36	F−: 0.073 ± 0.006	F+: 0.081 ± 0.006

Interaction terms of predictor variables were never significant and were removed from the models

Significant values (*P* < 0.05) highlighted bold

### Temporal dynamics

**Fig. 1:**
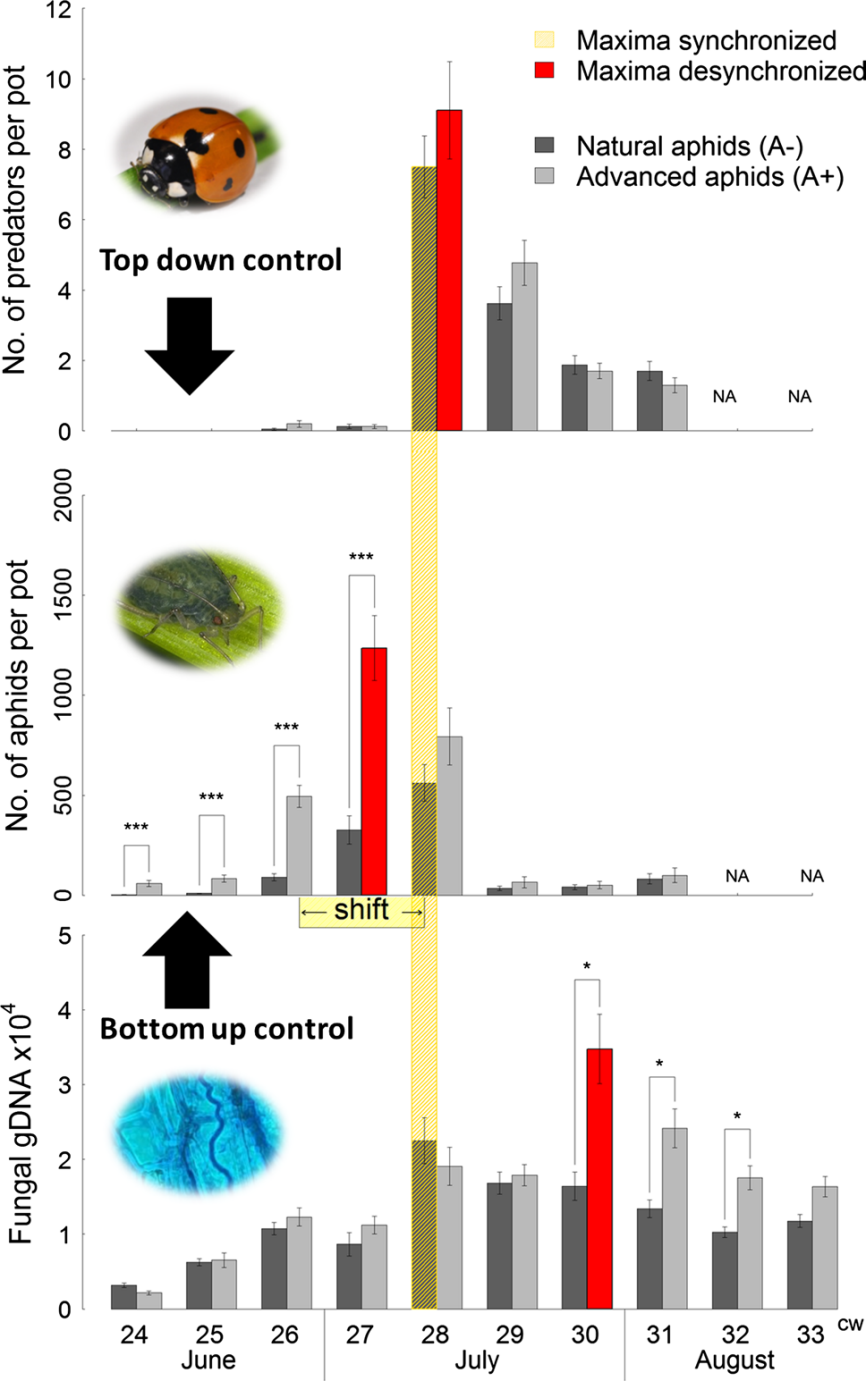
Temporal dynamics of aphids, predators, and fungal gDNA depending on aphid shift (A+, A−) over the study period. Fungal gDNA is only presented for endophyte infected pots. Mean ± S.E. per calendar week (cw) of aphid abundance, predator abundance, and concentration of fungal gDNA. *Yellow bar* synchronized maxima with naturally occurring aphids (A−), and *Red bars* desynchronized maxima with simulated aphid shift (A+). *←* *shift* *→*  Aphid abundance with natural aphid occurrence (A−) is similar to aphid abundance in enhanced aphid treatment (A+) 2 weeks earlier. *NA* not available, ***P ≤ 0.001, **P ≤ 0.01, *P ≤ 0.05

Aphid abundance increased in both A+ pots (aphid shift) and A− pots (no aphid shift) over the first weeks of the experiment (Fig. 1). Until calendar week 27 (first 4 weeks of the experiment), the weekly recorded aphid abundance was significantly higher in A+ than in A− pots. The aphid abundance in A+ pots in calendar week 26 corresponded approximately to the aphid abundance in A− pots reached in calendar week 28 indicating that the treatment A+ successfully simulated an advancement of aphid population growth by about 2 weeks (Fig. 1 “shift”).

Predators were nearly absent in both treatments until calendar week 27 (week four of the experiment). In that week, aphid abundance in A+ pots was three times as high as in A− pots (Fig. 1). Despite the advancement of the prey phenology in A+ pots by 2 weeks, predator abundances increased simultaneously in both treatments in calendar week 28 and reached similarly high values in A+ and A− pots (Fig. 1, Fig. S1). In other words, predators on A+ pots did not increase their abundance in parallel to their prey. With high predator abundances, the aphid abundances decreased within 2 weeks by more than 92% on all pots (Fig. 1).

**Fig. 2:**
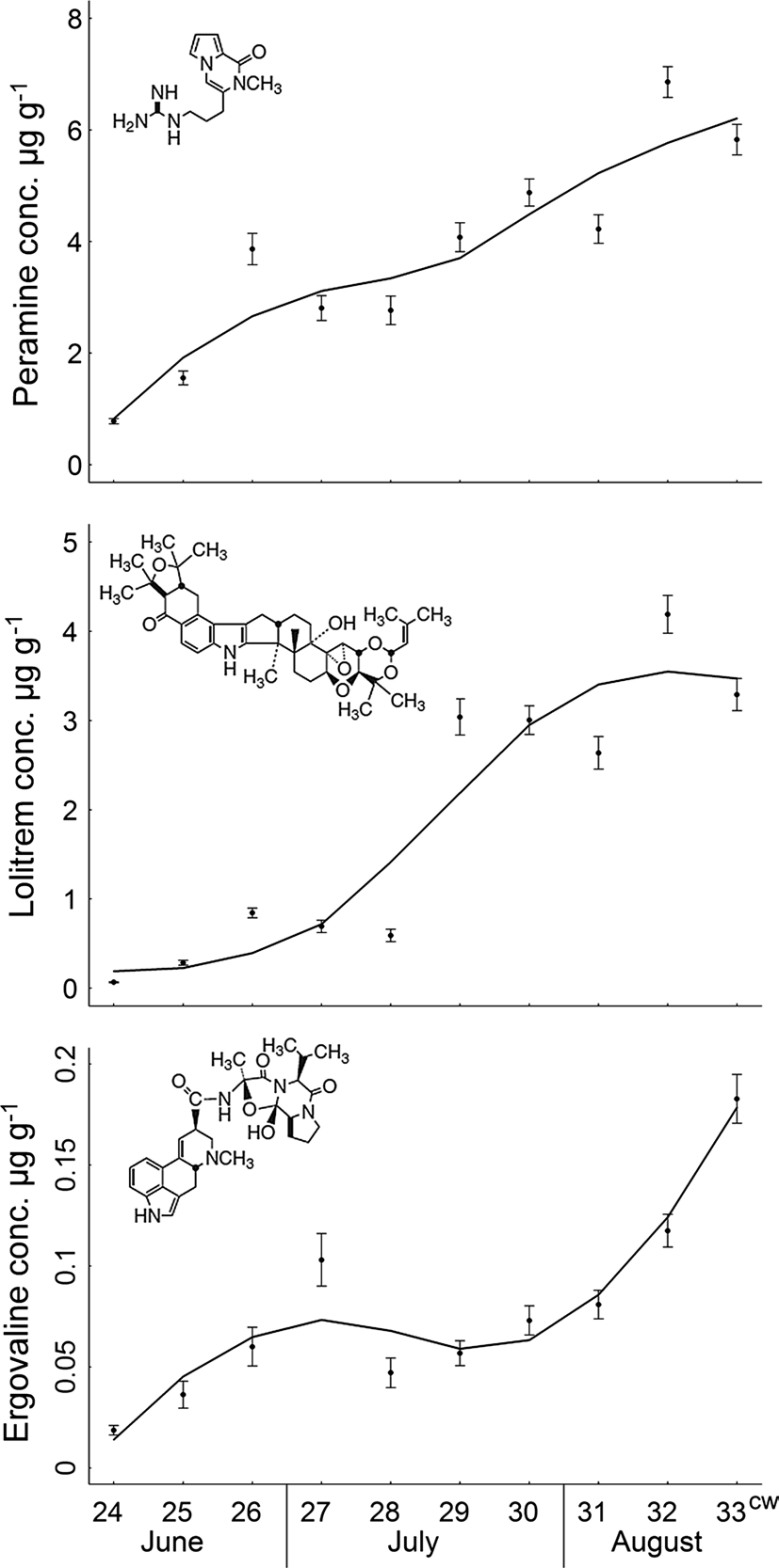
Change of alkaloid concentrations from late spring (cw 24) to summer (cw 33) in endophyte infected pots (*N* = 40). Mean ± S.E per calendar week (cw) of alkaloid concentrations in µg/g

Endophyte concentration in A− plants reached its maximum in calendar week 28 and was thus perfectly synchronized with the maximum of aphid abundances on the host plants (Fig. 1). Endophyte concentration in A+ plants reached its maximum in calendar week 30, which was 3 weeks after aphid abundance reached its maximum on the host plants. In calendar weeks 30–32, the endophyte concentration in A+ plants was higher than in A− plants. At this time, aphid populations were already controlled by top–down effects (predation and potentially emigration) and aphid abundances were very low in both treatments (Fig. 1). The alkaloids produced by the endophytic fungus did not significantly differ between A+ and A− plants during the study period, but increased in both treatments from mid-June until mid-August when we stopped recording concentrations (Fig. 2).

## Discussion

Enhanced aphid abundances in spring lead to overall higher aphid abundances, because neither aphid predators (top–down control) nor chemical defense of the endophyte (bottom–up control) had the plasticity to shift their phenology or to increase their abundance when aphid abundances reached high levels. However, as soon as predators occurred, aphid abundances dropped within 2 weeks, independently of aphid population size which displays the impact of predators in pest control. Endophyte concentration increased time delayed to enhanced aphid abundance at a time when bottom–up control had no effect on aphid control, because aphids were already top–down controlled. Phenology shift of aphids, therefore, caused desynchronization between the trophic levels resulting in (1) larger herbivore populations before top–down control by predators and (2) time-delayed bottom–up response by the endophytic fungus.

Increased aphid abundance in spring leads to overall higher aphid abundances and simulated a phenology shift of aphids. This result is not surprising, as aphids have a fast parthenogenetic life cycle (Leather and Dixon 1984) and the addition of aphid individuals allowed a larger starting population. We conclude that our experimental setting simulated a shift in aphid phenology of ~2 weeks, even though it remains difficult to separate effects between shift and abundance of aphids on interacting trophic levels. As top–down control by predators occurred temporally independent of aphid abundance, higher aphid abundances early in the season are likely to cause stronger plant damage (Van Emden and Harrington 2007).

We predicted that enhanced aphid abundance in spring leads to a temporal desynchronization with their predators (top–down control). We confirmed our prediction, because predators did not shift their phenology to an earlier arrival but occurred at the same time in both aphid treatments regardless of high differences in aphid abundances between the treatments. In contrast to a desynchronized caterpillar–bird system, where birds missed the peak abundance of their prey with possible fitness disadvantages for predators (Visser et al. 2006), aphid predators might even benefit from enhanced prey abundances. Surprisingly, plants with high prey abundances did not attract more predatory insects. Prevalent aphid predators were mostly larvae of hoverfly species, which choose their egg deposition sites by semiochemical cues, emitted by aphids and their host plants (Almohamad et al. 2007). We speculate that aphid predators were attracted by plant cues and general aphid presence rather than primarily distinguished between pots with higher or lower aphid abundance. If aphids occur earlier on plants, predators might as well arrive earlier, but their phenological plasticity is suggested to be lower than the one of aphids (Reed et al. 2011). With our study design, we cannot exclude that predator arrival can adapt to earlier aphid arrival, but our results indicate a plant quality and photoperiod triggered predator arrival. Plant biomass increased with fertilization in our study and higher predator abundances were recorded on fertilized pots which indicates that egg deposition is determined by plant cues rather than by aphid abundance. Further aphid predators occurred in 2 week of July with high abundances, presumably determined by photoperiod. Photoperiod triggers diapause and migration of aphid predators, such as hoverflies (Dingle 1972; Saunders 1981; Hondelmann et al. 2005), while the arrival of aphids is triggered by temperature (Zhou et al. 1995). Ongoing climate change increases temperature unlike photoperiod which indicates desynchronizations between aphids and their predators in future climate change scenarios. Even though we detected a desynchronization between aphids and aphid predators in our study, all aphid populations were top–down controlled within 2 weeks, highlighting predator efficiency in top–down controlling of aphid infestations (Symondson et al. 2002).

As predicted, the concentration of the endophytic fungus was increased in plants with enhanced aphid abundances in spring. However, endophyte concentration increased time delayed when aphid abundances were already controlled by predators and when no further herbivore species were present in considerable numbers. Defense strategies of plants and plant-associated symbionts can be costly and are often induced by prevalent herbivory (Strauss et al. 2002). Hosting the endophytic fungus can turn into a costly association for the plant when endophyte concentration increases following high herbivory but without affecting herbivory (Cheplick and Faeth 2009), especially when nitrogen is a limiting resource for plant growth and reproduction success competing with endophyte growth and alkaloid synthesis (Faeth and Fagan 2002). Our study shows evidence that enhanced herbivore abundance in spring can desynchronize the symbiotic relationship in a plant–endophyte association. This is in line with desynchronizations found in ant–aphid (Barton and Ives 2014), plant–mycorrhiza (Vega-Frutis et al. 2014), and plant–pollinator symbioses (Hegland et al. 2009).

The endophytic fungus-grass association used in our study produces different alkaloids which are toxic for herbivores (Clay 2014). We expected increased alkaloid concentrations on plants with higher aphid abundances, but our results showed similar alkaloid concentrations independent of aphid abundances. One explanation could be that alkaloids accumulate in the plant and alkaloid production might follow time delayed after endophytic growth. Another reason might be that alkaloid concentrations depend on the type of herbivory. Chewing herbivores and mechanical plant damage increased alkaloid concentrations in endophyte infected plants (Zhang et al. 2009; Fuchs et al. 2016), but plant sap sucking herbivores might not induce alkaloid production. Unlike earlier findings (Krauss et al. 2007), fertilization did not enhance endophyte and alkaloid concentrations, probably because soil used in our study already contained a high amount of nitrogen.

In contrast to our fourth prediction, aphid abundance was not significantly affected by the presence of the endophytic fungus (E+ vs E− plants). This is contrary to laboratory studies showing a reduced herbivore performance on host grass with an *Epichloë festucae* var. *lolii* symbiosis (Breen 1994; Meister et al. 2006), but is in accordance with no differences found in alkaloid concentration levels. An increase in endophyte concentration might be a first step towards induced bottom–up control following increased aphid abundance, thereby timing determines the endophytes mutualistic or antagonistic effects for the host plant. Nevertheless, endophyte growth was time delayed to high aphid abundance which strongly reduced the potential beneficial effect against herbivores, as herbivores were already leveled by top–down effects. Furthermore, the missing bottom–up control of aphids in our study might be caused by low alkaloid concentrations when aphid abundances peaked at the beginning of July. All three tested alkaloids increased over the study period probably due to accumulation in the plant. With an aphid shift to an earlier arrival in spring (Bell et al. 2015), an increasing temporal desynchronization between high aphid abundances in spring and high alkaloid concentrations in summer might be the consequence.

## Conclusions

We showed with our common garden experiment that enhanced herbivore abundance in spring affected interacting species in a multi-trophic system, which can desynchronize trophic cascades. In our multi-trophic level approach, the possible bottom–up control of herbivores (grass-associated microorganism) enhanced after herbivores had already been controlled by predators. Without the function of herbivore deterrence by the plant-associated microorganism, the mutualistic symbiosis could turn antagonistic with possible fitness costs for the symbiotic association which can alter the relations in interacting species (Saikkonen et al. 1998; Müller and Krauss 2005). Furthermore, we showed that aphid predators did not shift their phenology when aphid abundances were higher in early summer. Predator control of aphids was very efficient after the occurrence of predators. Nevertheless, plants were exposed to stronger herbivore pressure before the occurrence of predators. This can lead to higher plant damage and an increase in vector transmitted crop diseases (Fand et al. 2012). Our study indicates desynchronized predator–prey and plant–microorganism interactions within a food chain. Further research is needed to determine if similar effects occur among pests and their natural enemies in cropping systems under climate change.

## Electronic supplementary material

Below is the link to the electronic supplementary material.

Supplementary material 1 (DOCX 184 kb)

## References

[CR1] Almohamad R, Verheggen FJ, Francis F, Haubruge E (2007). Predatory hoverflies select their oviposition site according to aphid host plant and aphid species. Entomol Exp Appl.

[CR2] Barton BT, Ives AR (2014). Direct and indirect effects of warming on aphids, their predators, and ant mutualists. Ecology.

[CR3] Bell JR, Alderson L, Izera D (2015). Long-term phenological trends, species accumulation rates, aphid traits and climate: five decades of change in migrating aphids. J Anim Ecol.

[CR4] Bezemer TM, Jones TH, Knight KJ (1998). Long-term effects of elevated CO2 and temperature on populations of the peach potato aphid *Myzus persicae* and its parasitoid *Aphidius matricariae*. Oecologia.

[CR5] Breen JP (1994). Acremonium endophyte interactions with enhanced plant resistance to insects. Annu Rev Entomol.

[CR6] Chen M-S (2008). Inducible direct plant defense against insect herbivores: a review. Insect Sci.

[CR7] Cheplick GP, Faeth SH (2009). Ecology and evolution of the grass–endophyte symbiosis.

[CR8] Clay K (2014). Defensive symbiosis: a microbial perspective. Funct Ecol.

[CR9] Cleveland WS, Grosse E, Shyu WM (1992). Local regression models. Stat Models S.

[CR10] Costamagna AC, Van Der Werf W, Bianchi FJJA, Landis DA (2007). An exponential growth model with decreasing r captures bottom-up effects on the population growth of *Aphis glycines* Matsumura (Hemiptera: Aphididae). Agric For Entomol.

[CR11] Crawley MJ (2012). The R Book.

[CR12] de Sassi C, Müller CB, Krauss J (2006). Fungal plant endosymbionts alter life history and reproductive success of aphid predators. Proc R Soc B Biol Sci.

[CR13] Dingle H (1972). Migration strategies of insects. Science.

[CR14] Dixon AFG (1971). The life-cycle and host preferences of the bird cherry-oat aphid, *Rhopalosiphum padi* L., and their bearing on the theories of host alternation in aphids. Ann Appl Biol.

[CR15] Faeth SH, Fagan WF (2002). Fungal endophytes: common host plant symbionts but uncommon mutualists. Integr Comp Biol.

[CR16] Fand BB, Kamble AL, Kumar M (2012). Will climate change pose serious threat to crop pest management: a critical review?. Int J Sci Res Publ.

[CR17] Fuchs B, Krischke M, Mueller MJ, Krauss J (2013). Peramine and lolitrem B from endophyte–grass associations cascade up the food chain. J Chem Ecol.

[CR18] Fuchs B, Krischke M, Mueller MJ, Krauss J (2016). Herbivore-specific induction of defence metabolites in a grass–endophyte association. Funct Ecol Press.

[CR19] Gastal F, Nelson CJ (1994). Nitrogen use within the growing leaf blade of tall fescue. Plant Physiol.

[CR20] Härri SA, Krauss J, Müller CB (2008). Trophic cascades initiated by fungal plant endosymbionts impair reproductive performance of parasitoids in the second generation. Oecologia.

[CR21] Harrington R, Clark S (2010). Trends in the timings of the start and end of annual flight periods. Aphid biodiversity under environmental change.

[CR22] Hartley SE, Gange AC (2009). Impacts of plant symbiotic fungi on insect herbivores: mutualism in a multitrophic context. Annu Rev Entomol.

[CR23] Hegland SJ, Nielsen A, Lázaro A (2009). How does climate warming affect plant-pollinator interactions?. Ecol Lett.

[CR24] Hesse U, Schöberlein W, Wittenmayer L (2003). Effects of *Neotyphodium* endophytes on growth, reproduction and drought-stress tolerance of three *Lolium perenne* L. genotypes. Grass Forage Sci.

[CR25] Hondelmann P, Borgemeister C, Poehling H-M (2005). Restriction fragment length polymorphisms of different DNA regions as genetic markers in the hoverfly *Episyrphus balteatus* (Diptera: Syrphidae). Bull Entomol Res.

[CR26] IPCC WG III (2000). Special report on emissions scenarios.

[CR27] Kiers ET, Palmer TM, Ives AR (2010). Mutualisms in a changing world: an evolutionary perspective: mutualism breakdown. Ecol Lett.

[CR28] Koh S, Hik DS (2007). Herbivory mediates grass–endophyte relationships. Ecology.

[CR29] Krauss J, Härri SA, Bush L (2007). Effects of fertilizer, fungal endophytes and plant cultivar on the performance of insect herbivores and their natural enemies. Funct Ecol.

[CR30] Leather SR, Dixon AFG (1984). Aphid growth and reproductive rates. Entomol Exp Appl.

[CR31] Leuchtmann A, Bacon CW, Schardl CL (2014). Nomenclatural realignment of *Neotyphodium* species with genus *Epichloe*. Mycologia.

[CR32] Marquis M, Del Toro I, Pelini SL (2014). Insect mutualisms buffer warming effects on multiple trophic levels. Ecology.

[CR33] McCulley RL, Bush LP, Carlisle AE (2014). Warming reduces tall fescue abundance but stimulates toxic alkaloid concentrations in transition zone pastures of the U.S. Front Chem.

[CR34] Meister B, Krauss J, Härri SA (2006). Fungal endosymbionts affect aphid population size by reduction of adult life span and fecundity. Basic Appl Ecol.

[CR35] Müller CB, Krauss J (2005). Symbiosis between grasses and asexual fungal endophytes. Curr Opin Plant Biol.

[CR36] Parmesan C, Yohe G (2003). A globally coherent fingerprint of climate change impacts across natural systems. Nature.

[CR37] Rafferty NE, CaraDonna PJ, Burkle LA (2013). Phenological overlap of interacting species in a changing climate: an assessment of available approaches. Ecol Evol.

[CR38] Rasmussen S, Parsons AJ, Bassett S (2007). High nitrogen supply and carbohydrate content reduce fungal endophyte and alkaloid concentration in *Lolium perenne*. New Phytol.

[CR39] Reed TE, Schindler DE, Waples RS (2011). Interacting effects of phenotypic plasticity and evolution on population persistence in a changing climate: evolution, plasticity, and climate change. Conserv Biol.

[CR40] Rudgers JA, Clay K (2007). Endophyte symbiosis with tall fescue: how strong are the impacts on communities and ecosystems?. Fungal Biol Rev.

[CR41] Saikkonen K, Faeth SH, Helander M, Sullivan TJ (1998). Fungal endophytes: a continuum of interactions with host plants. Annu Rev Ecol Syst.

[CR42] Saunders DS (1981). Insect photoperiodism—the clock and the counter: a review. Physiol Entomol.

[CR43] Schardl CL, Florea S, Pan J (2013). The *Epichloae*: alkaloid diversity and roles in symbiosis with grasses. Curr Opin Plant Biol.

[CR44] Schmidt MH, Lauer A, Purtauf T (2003). Relative importance of predators and parasitoids for cereal aphid control. Proc R Soc Lond B Biol Sci.

[CR45] Snyder WE, Ives AR (2003) Interactions between specialist and generalist natural enemies: parasitoids, predators, and pea aphid biocontrol. Ecology 84:91–107. doi:10.1890/0012-9658(2003)084[0091:IBSAGN]2.0.CO;2

[CR46] Stevenson TJ, Visser ME, Arnold W (2015). Disrupted seasonal biology impacts health, food security and ecosystems. Proc R Soc B.

[CR47] Strauss SY, Rudgers JA, Lau JA, Irwin RE (2002). Direct and ecological costs of resistance to herbivory. Trends Ecol Evol.

[CR48] Symondson WOC, Sunderland KD, Greenstone MH (2002). Can generalist predators be effective biocontrol agents?. Annu Rev Entomol.

[CR49] Tanaka A, Tapper BA, Popay A (2005). A symbiosis expressed non-ribosomal peptide synthetase from a mutualistic fungal endophyte of perennial ryegrass confers protection to the symbiotum from insect herbivory: peptide synthetase protects symbiotum. Mol Microbiol.

[CR50] Thackeray SJ, Henrys PA, Hemming D (2016). Phenological sensitivity to climate across taxa and trophic levels. Nature.

[CR51] Tylianakis JM, Didham RK, Bascompte J, Wardle DA (2008). Global change and species interactions in terrestrial ecosystems. Ecol Lett.

[CR52] Van der Putten WH, Macel M, Visser ME (2010). Predicting species distribution and abundance responses to climate change: why it is essential to include biotic interactions across trophic levels. Philos Trans R Soc B Biol Sci.

[CR53] Van Emden HF, Harrington R (2007). Aphids as crop pests.

[CR54] Vega-Frutis R, Varga S, Kytöviita M-M (2014). Host plant and arbuscular mycorrhizal fungi show contrasting responses to temperature increase: implications for dioecious plants. Environ Exp Bot.

[CR55] Visser ME, Holleman LJM, Gienapp P (2006). Shifts in caterpillar biomass phenology due to climate change and its impact on the breeding biology of an insectivorous bird. Oecologia.

[CR56] Ward NL, Masters GJ (2007). Linking climate change and species invasion: an illustration using insect herbivores. Glob Change Biol.

[CR57] Zhang D-X, Nagabhyru P, Schardl CL (2009). Regulation of a chemical defense against herbivory produced by symbiotic fungi in grass plants. Plant Physiol.

[CR58] Zhou X, Harrington R, Woiwod ID (1995). Effects of temperature on aphid phenology. Glob Change Biol.

