# Supplementary material for: Enhanced aphid abundance in spring desynchronizes predator–prey and plant–microorganism interactions
Source: Oecologia. 2016 Nov 17;183(2):469–78. doi: 10.1007/s00442-016-3768-1 (PMC5306164; doi:10.1007/s00442-016-3768-1)
Supplement: Supplementary file 1 — Supplementary material 1 (DOCX 184 kb) [file 442_2016_3768_MOESM1_ESM.docx]

**Supplementary Material**

**Fig S1** Total number of aphids (a), average number of predators (b) and average concentration of fungal gDNA per grass DNA (c) shown for all pots (a,b) and only E+ pots (c). Values see Table 1. *** P≤ 0.001, ** P≤ 0.01, * P≤ 0.05

**Fig S1**

**
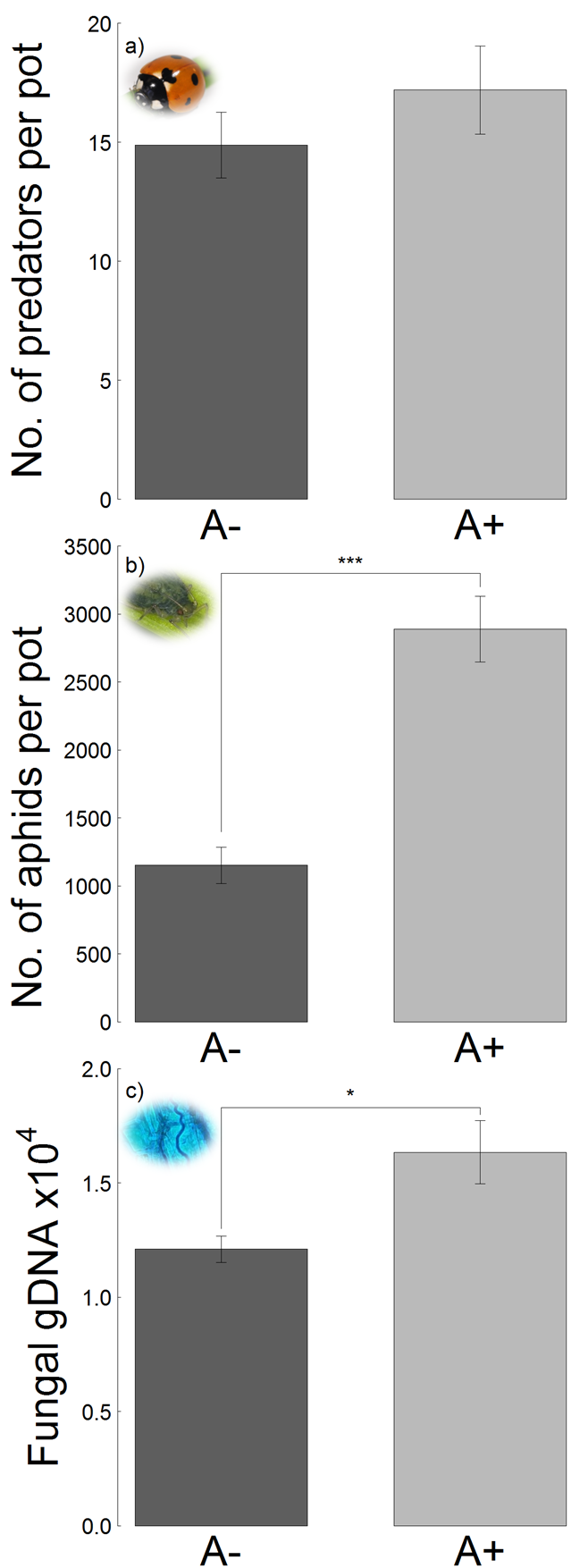
**

**Supplementary material to statistics:**

Code for analyzing **total differences** for every response variable (example aphid numbers). First we summed up the aphid numbers per pot (pot_id) over time:

**sum_pot_aphid_nr=aggregate(aphid_nr ~ pot_id+shift+endophyte+fertilizer, data=tatl_data, sum)**

Subsequently we conducted a linear model where we tested the effects of aphid shift (shift), endophyte infection (endophyte), fertilizer addition (fertilizer) and block (block) on aphid abundance. As block and interactions between the predictor variables were not significant, we simplified our model (m1k):

**m1k <- lm(aphid_nr~shift+endophyte+fertilizer, sum_pot_aphid_nr)**

**> anova(m1k)**

Code for analyzing **weekly differences** for every response variable with ANOVA of the linear model in a loop analysis (example aphid numbers):

**result_aphid<-list()for(i in 1:8){**

**result_aphid[[i]]<- anova(lm(aphid_nr~shift+endophyte+fertilizer,**

**tatl_data[tatl_data$week==i, ]))}**
